# Savoring Belief, Resilience, and Meaning in Life as Pathways to Happiness: A Sequential Mediation Analysis among Taiwanese University Students

**DOI:** 10.3390/bs14050388

**Published:** 2024-05-05

**Authors:** Der-Fa Chen, Kai-Wen Huang, Wei-Sho Ho, Yao-Chung Cheng

**Affiliations:** 1 Department of Industrial Education and Technology, National Changhua University of Education, Changhua 50007, Taiwan; dfchen@cc.ncue.edu.tw (D.-F.C.); kevinpopo@gs.slvs.tc.edu.tw (K.-W.H.); homaintain@gmail.com (W.-S.H.); 2 Center for Teacher Education, National Changhua University of Education, Changhua 50007, Taiwan

**Keywords:** savoring belief, happiness, resilience, meaning in life, sequential mediation model

## Abstract

In recent decades, scholarly interest has grown in the psychological components of happiness. Savoring belief, or the capacity to attend to, appreciate, and enhance the positive experiences in one’s life, along with resilience and meaning in life, have emerged as significant predictors of enhanced happiness among diverse populations. This research examined the interrelationships among savoring belief, resilience, meaning in life, and happiness. A sample of 561 students from 75 universities in Taiwan, comprising 361 female and 200 male participants with an average age of 20.88 years, participated in an online survey. The study employed various instruments, including the Savoring Belief Inventory, the Subjective Happiness Scale, the Brief Resilience Scale, and the Meaning in Life Questionnaire. These instruments were translated into Traditional Chinese using a back-translation method and subsequently validated for accuracy by specialists in the field. Analysis of the data using Hayes’ PROCESS Model 6 revealed several key insights: (1) savoring belief positively influenced happiness, resilience, and meaning in life with resilience further enhancing happiness and meaning in life; (2) resilience served as a significant mediator in the relationship between savoring belief and happiness; (3) meaning in life significantly mediated the relationship between savoring belief and happiness; (4) a sequential mediation model illustrated the mediating effects of resilience and meaning in life on the relationship between savoring belief and happiness. This study illustrates that, much like a garden requires water, sunlight, and care to flourish, our happiness is cultivated through enhancing our ability to savor the good moments, rebound from challenges, and find deep significance in our lives. We can significantly boost well-being by fostering these qualities—savoring belief, resilience, and a sense of meaning. These findings are particularly relevant for educators, highlighting the critical need to develop these traits in students to promote greater happiness and fulfillment in their lives. Discussions included theoretical implications, educational implications, and avenues for future research.

## 1. Introduction

Globally, cultivating high-quality university students is recognized as a pivotal strategy for augmenting national human capital [1]. Specifically, college-aged emerging adults navigate the complex transition to adulthood, facing various challenges across the human development spectrum, especially during early adulthood [2]. Students encounter numerous pressures and challenges throughout their university tenure, including academic stress, life transitions, and career planning. These elements can profoundly impact their mental and physical happiness, affecting their academic performance. Researchers have dedicated themselves to identifying psychological variables that significantly enhance happiness and aid in navigating life’s adversities. There have been efforts to examine disparities in happiness across nations [3] and predict happiness through advances in neurocognitive science [4]. Throughout history, happiness has fascinated philosophers, while contemporary psychologists have concentrated their research on strategies to improve individuals’ quality of life and happiness [5]. Happiness is defined in various ways, ranging from fleeting positive emotions to a long-term sense of meaning [6]. Some scholars suggest it encompasses an overall assessment of positive and negative emotions and life satisfaction [7]. Central to scholarly research is understanding factors contributing to enhanced happiness, such as savoring belief, resilience, and meaning in life.

Savoring the ability to focus on positive experiences and proactively enhance the quality of life by altering thoughts and behaviors has increased happiness [8]. Research suggests that savoring can serve as a beneficial intervention for managing worry and anxiety, leading to immediate increases in positive emotions and reductions in worry, particularly for individuals with Generalized Anxiety Disorder (GAD) [9]. Resilience, defined as the capacity to withstand stress and adversity, is a dynamic capability adaptable to environmental changes [9,10]. Studies have shown a positive correlation between resilience and happiness [11,12]. Resilience is a crucial resource for coping effectively with uncertainties and changes, such as during a pandemic, and involves emotional regulation and protective factors against psychological distress [13]. Support systems, including family and community, foster student resilience [14]. Meaning in life and finding purpose and significance through life experiences significantly influence happiness, self-esteem, hope, and self-efficacy [15,16]. Research indicates that meaning in life is inversely related to psychological distress, with resilience and loneliness mediating this relationship [17]. Meaning in life predicts university students’ psychological happiness, well-being, and happiness levels, and fulfilling needs for competence, autonomy, and relatedness is associated with life satisfaction through meaning in life [18,19].

Previous research has confirmed the correlation between university students’ savoring beliefs and happiness [20]. It has also been found that cultivating positive emotions can enhance students’ resilience, reduce negative emotions, and improve happiness [21]. Additionally, having a sense of meaning in life positively impacts the health of university students [22] and individuals with life goals tend to experience higher life satisfaction [23]. Research also indicates that savoring belief can increase positive emotions and reduce worry and anxiety. Resilience has been identified as a mediator in the relationship between meaning in life and psychological distress, suggesting that enhancing meaning in life can bolster resilience and reduce loneliness [17]. Recent studies have increasingly clarified the connections among savoring belief, subjective well-being, resilience, and meaning of life, particularly in higher education. Recent studies have explored the complex interactions between personal beliefs and psychological resilience, revealing several key insights. It has been demonstrated that social support and spirituality play crucial mediating roles in the relationship between resilience and quality of life among cancer survivors, underscoring the broader relevance of these constructs [24]. Similarly, introducing an allostatic active inference model has shed light on the role of resilience in well-being, emphasizing the importance of belief revision and action prioritization [25]. In religious contexts, the enhancement of resilience by spiritual and existential factors has been observed [26]. Additionally, the importance of optimism, hope, and spirituality as key predictors of well-being during crises has been identified, indicating their significance beyond merely aiding resilience [27]. These findings collectively highlight the interconnected nature of psychological constructs and their impact on improving life quality and resilience across various contexts.

Despite considerable research linking savoring belief to happiness, the intricate web connecting savoring belief, happiness, resilience, and meaning in life has not been thoroughly investigated. This gap signifies a missed opportunity to understand how these variables interact to shape students’ psychological landscapes. Our study addresses this deficiency by constructing a theoretical model that examines the potential influence of resilience and meaning in life on savoring beliefs and happiness among university students. By dissecting these relationships, this research seeks to enrich the academic discourse with a comprehensive framework and aspires to translate theoretical insights into practical strategies that could substantially enhance educational practices. The expected outcomes of this study include developing interventions that promote resilience and happiness, thereby contributing to more effective, evidence-based approaches in university settings.

## 2. Theoretical Foundation and Research Hypothesis Development

### 2.1. The Relationship between Savoring Belief and Happiness

The correlation between savoring belief and happiness has garnered extensive support from theoretical frameworks and empirical studies. Savoring belief is the ability to actively engage with the positive aspects of life’s situations whereas happiness involves satisfaction with one’s life and the consequent positive effects. Numerous studies have corroborated the positive relationship between savoring belief, life satisfaction, and happiness [20,28]. Furthermore, the enjoyment derived from savoring belief positive experiences has been identified as more significant than the frequency of such experiences [29]. Additionally, savoring belief has been shown to mitigate the impacts of adverse life events, such as stress, and mental health disorders, such as depression [30,31]. Recent research has highlighted savoring belief as a significant enhancer of positive emotions, happiness, and life satisfaction while reducing negative emotions [32]. Savoring belief also exhibits a strong association with psychological health [33], exerts a positive influence on the quality of intimate relationships [34], and can moderate the relationship between health-related pain and life satisfaction, ultimately leading to an elevated level of life satisfaction [33,35]. The reviewed literature forms the basis for the proposed hypothesis:

**Hypothesis** **1.** *Savoring belief positively predicts happiness.*

### 2.2. The Mediating Role of Resilience in the Relationship between Savoring Belief and Happiness

Resilience is an individual’s capacity for positive adaptation in adversity, influenced by personal traits and environmental factors, such as family and educational environments [36]. It comprises three interrelated components: adversity, outcomes, and mediating factors. Conceptually, resilience is a process leading to specific outcomes, with a significant focus on resilience research examining mediating processes. Highly resilient individuals can withstand considerable life stressors, such as poverty or family conflicts, without resorting to negative behaviors like violence or drug abuse. Instead, they often perceive challenges as opportunities for personal growth. Positive emotions contribute to enhancing resilience and happiness [37,38]. University students who possess high resilience are more adept at regulating the distress they encounter [39], effectively managing their emotions, reducing the incidence of depression [21], and experiencing reduced fatigue while learning challenging subjects. According to the poly-strengths theory (PST), resilience is a learnable skill that relies on individuals’ cognitive and behavioral strategies to overcome adverse events and enhance their happiness. Positive emotions, such as interest, happiness, satisfaction, and love, bolster individuals’ resilience [37]. Resilience has been shown to mediate the relationship between teacher support and adolescent mental health [40]. This literature review culminates in the proposition of the following hypotheses:

**Hypothesis** **2.** *Savoring belief positively predicts resilience.*

**Hypothesis** **3.** *Resilience positively predicts happiness.*

**Hypothesis** **4.** *Resilience mediates the relationship between savoring belief and happiness.*

### 2.3. The Mediating Role of Meaning in Life in the Relationship between Savoring Belief and Happiness

Meaning in life typically encompasses three components: cognitive, motivational, and emotional aspects [41]. According to the mindfulness-to-meaning theory (MMT) [42], mindfulness can enhance individuals’ emotional regulation abilities when faced with difficulties, promote positive emotions, and ultimately foster happiness, meaning in life, and psychological health [43] Research has consistently demonstrated the positive influence of meaning in life on the happiness of young individuals. Those with a higher sense of meaning in life tend to exhibit lower suicidal tendencies [22,44]. Additionally, the literature has indicated that meaning in life plays a mediating role in the relationship between social support and internet gaming addiction [45]. The use of meaning in life and self-efficacy as moderators has been suggested to potentially improve the subjective happiness of bullied adolescents [46]. Regarding elderly individuals, research has found that savoring belief contributes to a more positive mindset, subsequently enhancing life satisfaction [47].

Furthermore, a positive correlation has been identified between hope, trust, and subjective well-being, which in turn contributes to enhanced life satisfaction [48]. Research focusing on university students has discovered that higher life satisfaction is associated with a reduced occurrence of nonsuicidal self-injury behaviors. From the reviewed literature emerges the proposition of the following hypotheses:

**Hypothesis** **5.** *Savoring belief positively predicts meaning in life.*

**Hypothesis** **6.** *Meaning in life positively predicts happiness.*

**Hypothesis** **7.** *Meaning in life mediates the relationship between savoring belief and happiness.*

### 2.4. The Overall Relationship among Savoring Belief, Resilience, Meaning in Life, and Happiness

Resilience, characterized by adaptability and protective resources [13], is linked to enhanced happiness and life satisfaction [11,49]. Additionally, meaning in life is associated with psychological well-being and reduced distress, with resilience mediating this relationship [17]. Thus, resilience’s role in fostering adaptability and positive coping mechanisms likely contributes to an individual’s ability to find meaning in life, aligning with findings that resilient individuals experience greater life satisfaction and happiness, essential components of a meaningful life.

In exploring the relationship between savoring belief and happiness, it is essential to consider the sequential mediation of resilience and meaning in life. Savoring belief, identified as a factor that enhances happiness [47], sets the stage for this mediation process. The role of resilience, characterized by the ability to recover swiftly from adversities and promote mental health [50], becomes pivotal. Such adaptability is crucial for navigating stressful environments, influencing their happiness. Subsequently, the impact of meaning in life comes to the forefront. Moderated by positive emotions and psychological health [51], meaning in life significantly affects physical health and academic achievements [52,53]. The interplay between resilience and meaning in life is instrumental in connecting the savoring belief with enhanced happiness. This interplay aligns with the principles of Self-determination Theory (SDT), which posits that individuals actively seek challenges and new experiences [54]. Therefore, the combined effects of savoring belief, resilience, and meaning in life are critical in understanding how savoring belief influences overall happiness, highlighting their synergistic role in this mediation relationship. The building blocks of the literature pave the way for the proposition of the following hypotheses:

**Hypothesis** **8.** *Resilience positively predicts meaning in life.*

**Hypothesis** **9.** *Resilience and meaning in life sequentially mediate the relationship between savoring belief and happiness.*

### 2.5. The Present Research

**Figure 1:**
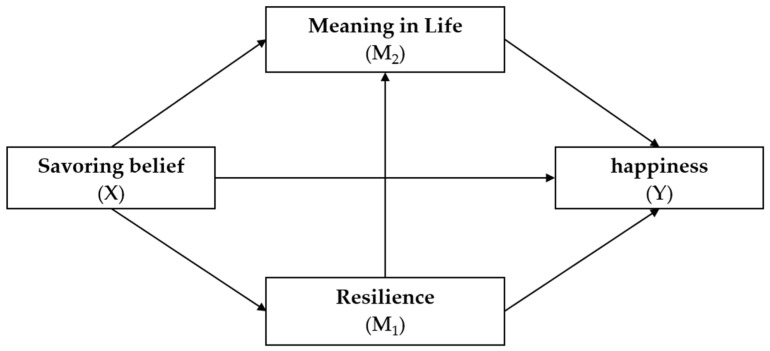
Hypothesized research model.

Based on the literature above, this research conducted an online questionnaire survey among university students in Taiwan to investigate the hypothesized model involving savoring belief, resilience, meaning in life, and happiness, as depicted in Figure 1.

## 3. Materials and Methods

### 3.1. Participants and Procedures

The present research focused on university students in Taiwan in 2022 as the target population. Convenience sampling, a nonprobability sampling method, was employed based on participants’ willingness, convenience, and accessibility [55]. The online questionnaire was disseminated through various channels, including university professors, educational communities, and course teaching platforms, from 25 April to 6 May 2022. This study conformed to the ethical principles of the 1975 Declaration of Helsinki, as revised in 2013, and met the local criteria for exemption from IRB review in certain social and behavioral research types. Written informed consent was secured from all participants, who were comprehensively informed about the study’s objectives, data utilization, anonymity of responses, and potential risks, thus adhering to the ethical standards required for non-interventional studies. Measures were implemented to ensure the anonymization of personal identifiers and the careful handling of identifiable information. Moreover, all participants voluntarily participated in the research and had no conflicts of interest with the researchers. According to the statistics provided by Taiwan’s Ministry of Education for 2022, there were 126 universities in Taiwan and 561 valid questionnaires were collected from 75 universities. Among the participants, 361 were females (64.4%) and 200 were males (35.6%). In terms of grade level, the distribution included 126 freshmen (22.4%), 134 sophomores (23.8%), 163 juniors (29%), and 138 seniors or above (24.8%). The age range of the students was 18 to 31 years old, with an average age of 20.88 years old.

### 3.2. Measures

This research employed well-documented scales in international academic journals and was often cited for measurement purposes. Five scholarly experts translated, reviewed, and refined these scales to ensure high content validity.

#### 3.2.1. Savoring Belief Inventory (SBI)

Savoring belief was assessed using the Savoring Belief Inventory (SBI) [56]. This scale comprises three dimensions: (1) Savoring the Future with four items (e.g., “Before a good thing happens, I look forward to it in ways that give me pleasure in the present.”), (2) Savoring the Present with three items (e.g., “I enjoy looking back on happy times from my past.”), and (3) Savoring the Past with three items (e.g., “I know how to make the most of a good time.”). Participants rated their responses on a 6-point Likert scale ranging from 1 (strongly disagree) to 6 (strongly agree). Higher scores indicated a stronger belief in savoring. The original scale demonstrated good internal consistency, with Cronbach’s α values of 0.86 for Savoring the Future, 0.84 for Savoring the Present, and 0.84 for Savoring the Past. In this research, the traditional Chinese version of the SBI had a good fit to the research data (χ^2^ = 272.199, *p* < 0.001, χ^2^/DF = 8.006, RMSEA = 0.112, GFI = 0.904, AGFI = 0.845, NNFI = 0.886, CFI = 0.914, IFI = 0.914), the overall Cronbach’s α for SBI was 0.92, with values of 0.822 for Savoring the Future, 0.808 for Savoring the Present, and 0.735 for Savoring the Past, indicating good internal consistency.

#### 3.2.2. Subjective Happiness Scale (SHS)

Happiness was evaluated by applying the Subjective Happiness Scale (SHS) [57]. This scale has been widely utilized in previous studies published in international journals [58], demonstrating good reliability and validity. The scale consists of 3 items (e.g., “In general, I consider myself: Not a very happy person/a very happy person.”), and participants rated their responses on a 7-point Likert scale ranging from 1 (strongly disagree) to 7 (strongly agree). Higher scores indicated higher levels of happiness. The original scale reported a Cronbach’s α of 0.86; in this research, the Cronbach’s α was 0.90, indicating good internal consistency.

#### 3.2.3. Brief Resilience Scale (BRS)

Resilience was evaluated through the application of the Brief Resilience Scale (BRS) [59]. This scale has been utilized in over 5000 studies and published in various international journals, indicating its robust reliability and validity [60]. The scale comprises six items (e.g., “I tend to bounce back quickly after hard times.”), with responses rated on a 5-point Likert scale ranging from 1 (strongly agree) to 5 (strongly disagree). Higher scores indicate higher levels of resilience. The original Cronbach’s α of the scale was 0.91, and in the present research, the Cronbach’s α was also 0.91, indicating good internal consistency. In this research, the traditional Chinese version of the BRS had a good fit to the research data (χ^2^ = 111.530, *p* < 0.001, χ^2^/DF = 12.392, RMSEA = 0.143, GFI = 0.934, AGFI = 0.845, NNFI(TLI) = 0.914, CFI = 0.948, IFI = 0.949), the overall Cronbach’s α for BRS was 0.901, indicating good internal consistency.

#### 3.2.4. Meaning in Life Questionnaire (MLQ)

Meaning in life was evaluated by applying the Meaning in Life Questionnaire (MLQ) [61]. This scale has been employed in several studies and published in international journals, demonstrating robust reliability and validity [62]. The scale consists of 9 items (e.g., “My life is arranged in a fairly organized way r”), with responses rated on a 6-point Likert scale ranging from 1 (strongly disagree) to 6 (strongly agree). Higher scores indicate a greater sense of meaning in life. The original Cronbach’s α of the scale was 0.62, whereas in this research, the Cronbach’s α was 0.88, indicating good internal consistency. In this research, the traditional Chinese version of the MLQ had a good fit to the research data (χ^2^ = 583.481, *p* < 0.001, χ^2^/DF = 21.610, RMSEA = 0.192, GFI = 0.799, AGFI = 0.664, NNFI(TLI) = 0.671, CFI = 0.753, IFI = 0.754,), the overall Cronbach’s α for MLQ was 0.862, indicating good internal consistency.

### 3.3. Statistical Analyses

A cross-sectional survey design was employed in this research, and data analysis and verification were conducted using SPSS 26.0 and the SPSS Hayes’ macro PROCESS v4.1. Preliminary analyses included descriptive analysis, Pearson product-moment correlation analysis, collinearity diagnostic analysis, and factor analysis. Subsequently, Model 6 was utilized to test the sequential mediation model between resilience and meaning in life, with savoring belief and happiness as mediators. The direct effects between each predictor variable and its outcome variable were analyzed, and the indirect effects of the hypothesized mediators between the predictor and outcome variables were examined to determine the nature of the relationships [63].

## 4. Results

### 4.1. Descriptive Statistics and Correlations

The skewness values for savoring belief, resilience, meaning in life, and happiness ranged from −1.631 to −0.031, and the kurtosis values ranged from −0.905 to 4.034, conforming to the criteria for normality as proposed by Kline (2016) [64]. Pearson correlation analysis was conducted to investigate the relationships among these variables. As shown in Table 1, the correlation coefficients among the variables were all below 0.85, indicating no severe multicollinearity [65]. Furthermore, the tolerance values in this research ranged from 0.651 to 0.864, and the variance inflation factor (VIF) values ranged from 1.214 to 1.536, suggesting no multicollinearity among the research variables [66].

Table 1 displays all research variables’ mean values, standard deviations, and correlations. Consistent with the literature review and research hypotheses, savoring belief exhibited a positive correlation with resilience (r = 0.235, *p* < 0.001), as well as positive correlations with both meaning in life and happiness (r = 0.419, *p* < 0.001; r = 0.526, *p* < 0.001). Moreover, resilience positively correlated with meaning in life and happiness (r = 0.504, *p* < 0.001; r = 0.517, *p* < 0.001). Meaning in life positively correlated with happiness (r = 0.698, *p* < 0.001). These findings from the correlation analysis supported the research hypotheses and provided a foundation for further investigation of the mediating effects among the variables.

### 4.2. Test the Extent of the Common Method Variance (CMV)

To assess the presence of common method variances (CMV), Harman’s single-factor test was employed to examine the unrotated exploratory factor analysis. The results indicated that the first factor accounted for 37.058% of the variance, which was less than 50%, suggesting the absence of common method variance (CMV) issues [67].

### 4.3. Testing the Sequential Mediation Model and Validating the Research Hypotheses

Before testing the mediation model, a regression analysis was conducted to explore the paths of influence among the research variables. As presented in Table 2 and Table 3, the total effect of savoring belief on happiness was significant (β = 0.525, *p* < 0.001), supporting Hypothesis 1. Savoring belief positively predicted resilience and meaning in life (β = 0.235, *p* < 0.001; β = 0.317, *p* < 0.001), thus supporting Hypothesis 2 and Hypothesis 5. Resilience positively predicted meaning in life and happiness (β = 0.428, *p* < 0.001; β = 0.212, *p* < 0.001), supporting Hypothesis 3 and Hypothesis 8. Meaning in life positively predicted happiness (β = 0.475, *p* < 0.01), supporting Hypothesis 6.

The integrated findings accentuate the substantial total effect of savoring belief on happiness (effect = 0.525, 95% CI [0.684, 0.897]), reinforcing the notion that the intrinsic ability to appreciate and savor positive experiences is foundational to an individual’s sense of well-being. The direct effect of savoring belief on happiness remains prominent (effect = 0.416, 95% CI [0.327, 0.505]), corroborating the premise that the capacity to savor transcends the mediating constructs to bolster happiness directly.

The mediation pathway delineates a significant indirect effect, initially through resilience (effect = 0.075, 95% CI [0.041, 0.115]), aligning with Hypothesis 4, and subsequently through meaning in life (effect = 0.227, 95% CI [0.169, 0.292]), upholding Hypothesis 7. Our analysis further validates Hypothesis 9, with the sequential mediation effect from savoring belief through resilience and meaning in life to happiness (effect = 0.072, 95% CI [0.044, 0.105]), thus confirming the full mediation model.

The sequential mediation highlights an intricate psychological cascade: savoring belief fortifies resilience, resilience, in turn, fosters a more profound sense of life’s meaning, and together, they elevate happiness. The indirect effects explicate that while savoring belief independently predicts happiness, its impact is magnified through the intermediary roles of resilience and meaning in life, offering a richer understanding of the interdependencies among these constructs.

### 4.4. Comparison of Differences in Gender, Grade, and Major on the Measures

Table 4 offers a comparative analysis across gender, grade level, and major subjects along four dimensions: savoring belief, happiness, resilience, and meaning in life. The study identified significant gender differences in mechanisms of psychological adaptability. Specifically, women exhibited higher scores on the Savoring Belief Inventory (SBI) with a mean (M) of 5.990 and a standard deviation (SD) of 0.822, surpassing men who scored an M of 5.833 (SD = 0.822). Conversely, men scored higher on the Brief Resilience Scale (BRS) with an M of 3.597 (SD = 0.823) compared to women who recorded an M of 3.224 (SD = 0.935), with t-values of 2.169, *p* < 0.05 and −4.735, *p* < 0.001, respectively, illustrating a significant gender impact on these adaptive processes. Nevertheless, gender differences were not significant on the Subjective Happiness Scale (SHS) and the Meaning in Life Questionnaire (MLQ) (SHS: t = 0.251, *p* > 0.05; MLQ: t = 0.727, *p* > 0.05).

Analysis regarding the impact of grade levels indicated no significant differences across the scales, suggesting that grade level does not significantly influence university students’ responses concerning savoring belief, happiness, resilience, and meaning in life (SBI: F = 1.565, *p* > 0.05; SHS: F = 1.537, *p* > 0.05; BRS: F = 1.554, *p* > 0.05; MLQ: F = 1.712, *p* > 0.05). This points to individual differences, rather than academic progression, influencing variations in psychological adaptability among college students.

Further examination of the influence of students’ major subjects revealed no statistically significant differences in the measured dimensions (SBI: t = −0.688, *p* > 0.05; SHS: t = −0.744, *p* > 0.05; BRS: t = 1.499, *p* > 0.05; MLQ: t = −1.782, *p* > 0.05). These results imply that differences in psychological adaptability among university students are driven by individual difference factors rather than their field of study, highlighting the complex interplay of personal characteristics in psychological adaptation within higher education.

## 5. Discussion

### 5.1. Investigating the Positive Correlation between Savoring Belief and Happiness

**Figure 2:**
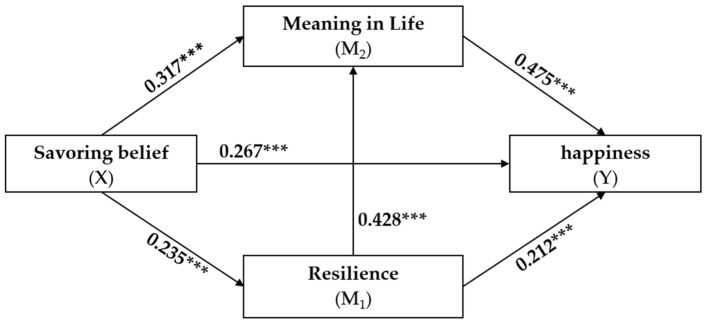
The sequential mediation model of this research. *** *p* < 0.001.

The sequential mediation model of this research is illustrated in Figure 2. The model confirms a positive link between savoring belief and happiness, aligning with existing studies [68]. The literature consistently showed that savoring belief positively affected happiness across different demographics [34] and reduced negative emotions such as stress and depression [31]. Savoring beliefs are increasingly acknowledged for their capacity to enhance positive emotions and alleviate worries and anxiety immediately. Directed savoring activities deliver significant emotional advantages compared to tasks lacking a savoring component. This emphasizes the role of savoring in breaking cycles of worry and fostering emotional resilience, which are crucial for enhancing students’ well-being. Integrating savoring strategies into mental health interventions in educational settings could significantly benefit student outcomes. This investigation confirmed that university students could improve their happiness by fostering savoring belief capabilities.

### 5.2. Exploring the Mediating Role of Resilience in the Relationship between Savoring Belief and Happiness

The findings aligned with Hypothesis 4, demonstrating resilience’s significant mediating role between savoring belief and happiness, echoing insights from resilience theory (RT) and poly-strengths theory (PST). As both a process and an acquirable skill, resilience enables individuals to overcome adversity and enhance happiness. This research solidified resilience’s mediating role in the savoring belief-happiness nexus among university students, a notion supported by prior work [12]. Additionally, Savoring belief is a potent intervention for elevating positive emotions and alleviating anxiety, thus bolstering happiness. Scholars have elucidated the protective role of resilience against psychological distress, underscoring its value within strategies for confronting adversity [13]. These findings highlight the integral role of resilience in psychological happiness.

### 5.3. Examining the Mediating Role of Meaning in Life in the Savoring Belief-Happiness Nexus

This research sought to explore the pathway delineated in Hypothesis 7, and the findings corroborated that meaning in life acts as a mediator in the relationship between savoring and well-being. Meaning in life functions as a mechanism that not only mitigates the experience of negative emotions but also fosters positive emotions, such as satisfaction and happiness, thereby bolstering the resilience of university students in the face of adversity [69]. Additionally, research by Wolfram (2023) explored the pivotal role of meaning in life as a mediator in enhancing well-being through savoring, indicating the importance of meaning in life in mental health interventions for university students [17].

### 5.4. Assessing the Sequential Mediation of Resilience and Meaning in Life in the Context of Savoring Belief and Happiness

The sequential mediation model demonstrated that resilience and meaning in life sequentially mediated the relationship between savoring and well-being, supporting Hypothesis 9. Prior research involving university students has demonstrated that resilience markedly enhanced well-being [70], with further studies evidencing a positive association between meaning in life and favorable outcomes, including positive emotions and academic success [71]. Savoring has been identified as a factor that augments resilience, culminating in heightened well-being and life satisfaction [49]. These results aligned with and bolstered the findings of related research.

### 5.5. Analyzing the Impact of Savoring Belief on Happiness: Differences in Gender, Grade, and Major

This study examined psychological adaptability among university students, focusing on the impacts of gender, grade levels, and majors across four dimensions: savoring belief, happiness, resilience, and meaning in life. It identified significant gender differences in specific adaptability mechanisms: women exhibited higher savoring beliefs, whereas men demonstrated greater resilience. These findings highlight a distinct gender influence on these adaptive processes.

No significant differences were found in subjective happiness or meaning in life related to gender. Similarly, the study revealed that grade level had a minimal impact on all psychological adaptability dimensions, indicating that academic progression does not substantially influence these traits.

Further analysis of the influence of students’ majors also indicated no significant differences, suggesting that individual differences, rather than academic or disciplinary factors, predominantly drive psychological adaptability. This underlines the complex role of personal characteristics in the psychological adaptation of college students within the higher education setting.

In summary, this research validated a sequential mediation model, which outlined six direct and three indirect paths, supporting the sequential mediating roles of resilience and meaning in life between savoring belief and happiness.

## 6. Implications

### 6.1. Theoretical Implications

This research presented several theoretical implications. Firstly, it was found that increased savoring belief among university students enhanced their happiness, corroborating the mindfulness-to-meaning theory (MMT) posited by Garland et al. (2015) [42]. This theory delineates a positive psychological cycle encompassing decentering, attentional broadening, reappraisal, and savoring belief [72]. It suggests that mindfulness during adversity helps construct meaning and personal growth, influencing happiness.

Secondly, this research demonstrated that resilience strengthens the relationship between savoring belief and happiness; individuals with higher resilience enhanced their happiness more effectively through savoring belief. This finding supports Kumpfer’s resilience framework, which indicates the mediating role of resilience between stress and outcomes [73]. It suggests that enhancing resilience after facing difficulties can improve happiness, with support systems such as family and community playing a pivotal role [19].

Thirdly, the findings revealed that perceiving a high meaning in life enhances the relationship between savoring belief and happiness. Supporting the self-determination theory proposed by Deci and Ryan (1985), the satisfaction of psychological needs—autonomy, competence, and relatedness—links meaning in life to happiness [54,74]. Understanding the intrinsic motivations of university students elucidates the environmental and societal influences [75].

Finally, the research confirmed savoring belief’s impact on happiness and identified significant mediating effects of resilience and meaning in life in the happiness model. This underscores the importance of understanding and enhancing university students’ happiness, laying a foundation for educational and psychological interventions.

### 6.2. Educational Implications

The educational implications of this research are as follows. Firstly, the research discovered that happiness, previously recognized to benefit from savoring belief, can be further enhanced by nurturing the meaning of life and resilience. Individuals with high resilience were found to withstand significant life stress, recover swiftly during hardships, and reduce the risks of mental illnesses [51,76]. Resilience, a skill that can be learned, can be strengthened by positive emotions such as interest, joy, satisfaction, and love [49,55]. Additionally, a strong sense of life’s meaning correlated with lower suicidal tendencies and a more optimistic view of life [77]. As students recognize the value of life’s meaning through achievements in overcoming challenges, fostering a positive outlook can enhance happiness and build resilience against adversity. This aligns with findings that resilience and positive affect, supported by teacher and peer encouragement, indirectly improve perceived academic performance, underscoring the role of emotional regulation, and resilience as protective factors during stressful periods [13].

Secondly, educational institutions play an essential role in stress management for students. University counselors can guide students to recall and cherish pleasant experiences, thereby enhancing savoring belief and mental health. Schools might organize activities to develop mental strength, teach students how to strengthen resilience, discover the meaning of life, and improve overall mental wellness. Emphasizing positive psychology enables students to enhance their happiness, psychological resilience, and relationships.

Finally, parental involvement in monitoring university students’ mental health, promoting positivity in adversity, and closely collaborating with schools is crucial in fostering students’ mental happiness. Educational authorities, schools, families, and community organizations should collaborate to provide diverse support, aiding university students in fully realizing their potential for an improved quality of life [14].

## 7. Limitations and Future Research Scopes

This research identified limitations. The sample was limited to Taiwanese university students. Future studies should expand to various age groups, educational systems, and cultures to enhance the findings’ universality. Although current models and scales assessed some variables, they might need to fully capture students’ psychological experiences, possibly affecting result validity. Future research should include qualitative methods to explore deeper motivations and psychological mechanisms. Employing a cross-sectional design may limit understanding of causal relationships [78]; longitudinal designs in future work could clarify these associations. This research focused on resilience and meaning in life as mediators. Including variables like dispositional mindfulness, personality traits, and positive psychological capital in future research could provide a more comprehensive analysis [79,80].

## 8. Conclusions

This research aimed to explore the relationship between savoring belief and happiness among university students and the sequential mediating roles of resilience and meaning in life in this relationship. The findings revealed that university students who exhibited a higher degree of savoring belief also displayed higher happiness. Resilience was found to significantly mediate between savoring belief and happiness. Furthermore, meaning in life also acted as a substantial mediator between savoring belief and happiness, further enhancing the positive impact of savoring belief on happiness. In the sequential mediation model, resilience and meaning in life demonstrated significant sequential mediating effects between savoring belief and happiness. This research highlighted the importance of savoring belief, resilience, and meaning in life to augment an individual’s happiness. This research demonstrates that just as a thriving garden needs water, sunlight, and careful tending, our happiness grows when we improve our capacity to enjoy positive experiences, recover from setbacks, and discover profound meaning in our existence. We can greatly enhance our happiness by nurturing these attributes—appreciation of life’s pleasures, resilience, and a deep sense of purpose. The results are especially significant for educators, underscoring the importance of cultivating these characteristics in students to foster increased happiness and satisfaction in their lives. Moreover, the research provided practical implications for the field of education, stressing the need to cultivate students’ savoring belief, resilience, and the ability to find meaning in life, thereby improving their happiness. Future research should examine additional potential factors influencing happiness and evaluate the generalizability of the findings across different cultural contexts and age groups.

## Figures and Tables

**Table 1:** Descriptive statistics and correlations for all variables (N = 561).

	Mean	SD	(1)	(2)	(3)	(4)	Skewness	Kurtosis
(1) savoring belief	5.937	0.892	1				−0.802	0.578
(2) resilience	3.376	0.948	0.235 ***	1			−0.296	−0.232
(3) meaning in life	3.430	0.726	0.419 ***	0.504 ***	1		−0.418	0.171
(4) happiness	4.737	1.342	0.526 ***	0.517 ***	0.698 ***	1	−0.566	0.210

*** *p* < 0.001.

**Table 2:** Regression analysis results (N = 561).

Regression		Model Index		Coefficients	
Outcome Variables	Independent Variables	R^2^	F	β	t
resilience	savoring belief	0.050	37.792 ***	0.235	5.726 ***
meaning in life	savoring belief	0.349	149.610 ***	0.317	9.045 ***
	resilience			0.428	12.201 ***
happiness	savoring belief	0.587	264.080 ***	0.276	9.231 ***
	resilience			0.212	6.727 ***

Note: *** *p* < 0.001, β = standardized coefficients. All variables were standardized.

**Table 3:** Total, direct, and indirect effects of savoring belief on happiness (N = 561).

Paths	Full	
	Effect	95% CI
Total effect	0.525	[0.684, 0.897]
Direct effects	0.416	[0.327, 0.505]
Indirect effect		
Total indirect effects	0.374	[0.295, 0.461]
savoring belief → resilience →happiness	0.075	[0.041, 0.115]
savoring belief → meaning in life →happiness	0.227	[0.169, 0.292]
savoring belief → resilience → meaning in life →happiness	0.072	[0.044, 0.105]

Note: Bootstrap sample size = 5000. CI = confidence interval.

**Table 4:** Differences in measure scores among the research sample based on demographic variables. (N = 561).

Demographic Variables	N	Savoring Belief Inventory (SBI)			Subjective Happiness Scale (SHS)			Brief Resilience Scale (BRS)			Meaning in Life Questionnaire (MLQ)		
		M	SD	F/t	M	SD	F/t	M	SD	F/t	M	SD	F/t
Gender 1. Female 2. Male	358 203	5.990 5.833	0.822 0.822	2.169 *	4.713 4.685	1.239 1.348	0.251	3.224 3.597	0.935 0.823	−4.735 ***	3.417 3.373	0.669 0.691	0.727
Grade 1. Freshman 2. Sophomore 3. Junior Year 4. Senior Year	121 128 158 154	5.798 5.984 5.935 5.997	0.862 0.677 0.630 0.641	1.565	4.490 4.709 4.785 4.782	1.360 1.213 1.238 1.298	1.537	3.252 3.303 3.474 3.371	0.856 0.942 0.960 0.878	1.554	3.306 3.358 3.471 3.439	0.737 0.638 0.641 0.688	1.712
Major 1. A 2. B	209 352	5.902 5.952	0.790 0.846	−0.688	4.651 4.734	1.268 1.284	−0.744	3.434 3.314	0.905 0.917	1.499	3.335 3.440	0.693 0.665	−1.782

Note: *** *p* < 0.001, * *p* < 0.05; A: natural sciences, engineering, and medical fields; B: humanities, social sciences, business management fields, other fields, and miscellaneous fields.

## Data Availability

Upon request, the data underpinning the results of this research can be obtained from the corresponding author.
